# Baltimore College of Dental Surgery

**Published:** 1845-03

**Authors:** 


*Baltimore College of Dental Surgery.*—This institution closed its regular annual course of lectures, comprising four months, the latter part of February last; and it may be gratifying to those who have been watching its progress, from the first moment of its inception to the present time, and who have regarded its success a very doubtful contingency, and its very existence, consequently, of very brief duration; we say, it gives us pleasure to inform such friends, who have had such anxieties and doubts, though in all kindness, sincerity and good will, that they may now dispel them forever, and regard the question of the existence, continuance, and future success of the college, as no longer a matter of experiment, but, henceforth, settled; and, further, satisfactorily settled.

It may not be amiss here to state a fact or two, as the grounds of such decision and encouragement.

*First.* The dental, and, we may add, the medical profession, as well as those from among the people at large, have acknowledged its claims by becoming pupils, amounting in all to the number of about forty-five, since the college first went into operation. Of this number, some were regular graduates of medical schools, and others had long practised, and attained to no inconsiderable eminence in their profession. About twenty have received the honors of the college, and are now practising in different and distant states of the union. From these the voluntary statement, and honest belief, has been expressed, that more real and useful information, in the science and art of dentistry, can be acquired by one course of instruction in the college, than can be possibly gained in four or five years' study in the private office of any dentist. Let it be recollected, however, that two courses of lectures are required to bring candidates forward for graduation; for one of which, a course in any respectable medical school is received as equivalent. This fact, consequently, proclaims the great utility and superior facilities of the dental college, for imparting a more sound, thorough and practical knowledge of the dental art, than can be obtained in any other way, and may be supplied at less than one-half the cost.

This is surely ground of encouragement, not only to the faculty, but to the profession at large; the whole community, and to every one, who, at heart, desires the elevation, the dignity, and usefulness of the science, together with the preservation of the health and lives of his fellow-beings.

*Second.* The approval and countenance of the various medical journals throughout the country, so far as any opinion has been expressed, is another ground of encouragement, and the last fact we shall notice under this head is one of very recent occurrence, to wit, the granting of a charter by the legislature of Ohio for the establishment of a similar college. This surely speaks volumes of encouragement, for here we find states are taking hold of the matter; that the people are publicly proclaiming through their legislatures that their wants and necessities need and must have such institutions, to shield them from the ignorance, the impudence, the boldness, and consequent injury, by the unblushing effrontery of dental quackery, that has been and is still stalking with more than giant strides throughout the length and breadth of our land.

Maryland has the credit of first starting in this matter, by establishing the Baltimore College of Dental Surgery of which we are speaking, and which has been in full operation for five years.

To those who are not so familiar with this college, as to witness its character and facilities, it may be necessary to state, in brief, that there are four professorships; comprising anatomy and physiology, special pathology and therapeutics, practical dentistry, and dental physiology and pathology. There is also a complete and commodious dental workshop, where the student is constantly engaged in practising his eyes and hands in the various manipulations of his art. The college has likewise a dissecting room, where the opportunity is furnished, and strongly urged upon the student, to use the knife in investigating all those organs, a knowledge of which is so necessary in all his operations upon the living subject, and which cannot be properly acquired without dissection.

We should also mention the college museum, as containing many valuable specimens of anatomy, some of which are from Paris, (and others are ordered, which will arrive before the commencement of the next course of lectures,) and morbid preparations of the teeth in all their different varieties and disease. Likewise the dental library, belonging to the professor of practical dentistry, being the most choice and extensive in this country, if not in the world.

We shall not dwell longer on the facilities of this institution, as all these points, will be more fully noticed in the annual circular soon to be issued; and we will close, by simply remarking, that the faculty, although provided with an ample building for their purpose, still, as this is only temporary, intend erecting one as soon as possible, on a plan commensurate with, and solely adapted to all the objects of such an institution.

